# Aberrant Origin of Vertebral Artery and its Clinical Implications

**DOI:** 10.5935/1678-9741.20150071

**Published:** 2016

**Authors:** Shi-Min Yuan

**Affiliations:** 1 The First Hospital of Putian, Teaching Hospital, Fujian Medical University, Putian, Fujian Province, China.

**Keywords:** Neurologic Manifestations, Vertebral Artery, Vertebral Artery Dissection

## Abstract

Aberrant origin of vertebral artery is rare. The anatomical features and clinical significance of this lesion remain to be clarified. A comprehensive collection of the pertinent literature resulted in a cohort of 1286 cases involving 955 patients and 331 cadavers. There were more left than right and more unilateral than bilateral aberrant vertebral arteries. Patients with aberrant origin of vertebral artery were often asymptomatic and in only 5.5% of the patients their symptoms were probably related to the aberrant origin of vertebral artery. The acquired cardiovascular lesions were present in 9.5% of the patients, 20.9% of which were vertebral artery-associated lesions. Eight (0.8%) patients had a vertebral artery dissection. Logistic regression analysis showed significant regressions between bovine trunk and left vertebral artery (*P*=0.000), between the dual origins of vertebral artery and cerebral infarct/thrombus (*P*=0.041), between associated alternative congenital vascular variants and cervical/aortic dissection/atherosclerosis (*P*=0.008). Multiple logistic regression demonstrated that side of the aberrant origin of vertebral artery (left vertebral artery) (*P*=0.014), arch branch pattern (direct arch origin) (*P*=0.019), presence of the common trunk (*P*=0.019), associated acquired vascular disorder (*P*=0.034) and the patients who warranted management (*P*=0.000) were significant risk predictors for neurological sequelea. The patients with neurological symptoms and those for neck and chest operations/ interventions should be carefully screened for the possibility of an aberrant origin of vertebral artery. The results from the cadaver metrology study are very helpful in the design of the aortic stent. The arch branch pattern has to be taken into consideration before any maneuver in the local region so as to avoid unexpected events in relation to aberrant vertebral artery.

**Table:** 

**Abbreviations, acronyms & symbols**	
QUOROM	= Quality of Reporting of Meta-Analyses
VA	=Vertebral artery

## INTRODUCTION

The vertebral artery (VA), which usually arises from the posterosuperior aspect of the first part of the subclavian artery and enters into the intracranial space via the dura mater at first cervical vertebra (C) and reaches C6 after traveling through the *foramen transversarium*, is an important blood supply of the brainstem and cerebellum^[1]^. VA pathologies, including anomalous origin and course, dual arteries, duplication, fenestration, tortuosity, elongation, kinking, arachnoid cysts, aneurysmal formation and associated hereditary connective tissue disorder, implicate typically in cerebrovascular events as a source of blood supply of posterior circulation^[2]^. Steal syndromes can be present in the condition of certain situations characterized by VA flow inversion^[3]^. Aberrant origin of VA is a rare variant of VA pathologies implicating in not only cerebrovascular events, but also VA dissection^[4]^ and surgical anatomy of local regions in particular in the operation of the carotid artery^[5]^ or aortic arch^[6]^. The "vertebral arteria lusoria", although even more rarely seen, should be considered in the patients undergoing esophageal surgery, and unawareness of such an aberrant VA may cause life-threatening events^[7]^. Moreover, dual origins of VA should be recognized in preoperative evaluation of patients with extracranial vascular disease^[8]^. This article aims to highlight the clinical importance and surgical anatomy of the aberrant origin of VA.

## METHODS

Publications in English language reporting on aberrant VA until February 2015 were retrieved in MEDLINE, Highwire Press and Google search engines. The search terms "aberrant origin", "dual/duplicated/bifid origins", "vertebral artery" and "aortic arch branching" were searched.

Primary exclusion criteria included articles describing aberrant origin of VA without giving patient number, other types of lesions of VA than aberrant origin. Studies with no complete data were excluded for the pertinent statistical analyses. Data were carefully extracted for details of the patient population, demographics, clinical symptoms, characteristics of VA, aortic branching pattern, common trunk of the artery, entry of VA into the *foramen transversarium*, associated congenital/acquired vascular disorders, associated otherwise disorders and cerebral events, *etc.* This rare condition was mostly reported in sporadic single cases or small series while a few with larger patient population. Accordingly, the qualitative analysis of the collective data from the retrieved articles constituted a systematic review, as suggested in the Quality of Reporting of Meta-Analyses (QUOROM) recommendations.

The study subjects were divided into two groups, cadaver and patient groups. The anatomy study involved both groups; while the clinical study was performed solely in the patient group.

Quantitative data were presented as mean ± standard deviation along with range and median values, and intergroup differences were compared by unpaired *t*-test. Comparisons of frequencies were performed by Fisher's exact test. *P*<0.05 was considered statistically significant.

## RESULTS

The literature retrieval generated a total of 214 articles with 1286 cases involving 955 (74.3%) patients and 331 (25.7%) cadavers.

There were 345 (58.5%) males and 245 (41.5%) females of the studying subjects whose gender was given. The gender ratio was 1.41. The patients' age was 48.2±21.0 (range, 0-89; median, 51) years (*n*=130) and the cadaver's' age was 57.4±23.4 (range, 0-94; median, 61) years (*n*=46).

The presenting symptoms or causes for presentation were described in 168 (17.6%) individuals of the patient group. Five (3.0%) patients were asymptomatic and 163 (97.0%) were symptomatic. Of the latter, the symptoms were associated probably with the lesions of the aberrant VA in 9 (5.5%) (Table 1), and due to lesions other than aberrant VA (including cerebral hemorrhage, cervical arterial disorder, post-traumatic syndrome, or aortic dissection) in the remaining 154 (94.5%) patients.

**Table 1:** Symptoms related to aberrant vertebral artery disorder in 9 patients.

Symptom	Disorder of aberrant VA	Site of aberrant VA
Headache	Aneurysm	Left VA
Vertigo, weakness, light-headedness	Critical stenosis	Left VA origin
Vertigo, cervicalgia	Dissection	Right VA (duplicated)
Occipitalgia	Dissection	Left VA
Neck pain, vertigo, vomiting, left facial droop and unsteady gait	Dissection	Left VA
Dizziness	Fenestration	Left VA
Headache	Hypoplasia	Right VA
Dizziness, headache	Kink	Right VA (dual origins)
Vertigo, weakness, nausea	Thrombus	Left VA origin

VA=vertebral artery

There were more left than right and more unilateral than bilateral aberrant VAs (Table 2).

**Table 2:** The aberrant vertebral artery in 1286 cases.

Aberrant VA	n (%)
Single aberrant origin	1233 (95.9)
Left VA	1056 (85.6)
Right VA	144 (11.7)
Bilateral VA	33 (2.7)
Dual aberrant origin	53 (4.1)
Left VA	30 (56.6)
Right VA	16 (30.2)
Dual aberrant right VA + single aberrant left VA	3 (5.7)
Bilateral dual origins	4 (7.5)

VA=vertebral artery

The single aberrant origin and dual origins of VAs were depicted in Tables 3 and 4. Hypoplastic VAs were found in 28 (2.2%) cases. Abnormal course of the aortic arch branches was found in 31 (2.4%) cases, with aberrant right VA being the most common (60%). The fusion level of the dual origins of the VAs was reported in 20 (1.6%) patients for 21 pairs of VAs, of which the left VAs fused most frequently at C5-6 (33.3%). Level of entry of the VAs into the *foramen transversarium* were expressed for 100 left and 33 right VAs with most of the left VAs entering into C5 (43%) and most of the right entering into C7 (21.2%).

**Table 3:** Single abnormal origin of vertebral arteries in 1231 cases.

Abnormal origin	n (%)
Left VA	980 (84.7)
Arch	955 (97.4)
Between LC & LS	782 (81.9)
Between RC + LC & LS	71 (7.4)
Between RS & RC + LC	1 (0.1)
Behind LC	2 (0.2)
Distal to LS	37 (3.9)
Posterior to the origin of LS	1 (0.1)
LS root	46 (4.8)
VA + LS (1 behind LC)	15 (1.6)
Extra-arch	5 (0.5)
Left external carotid artery	2 (40)
Thyrocervical trunk	1 (20)
Carotid bulb	1 (20)
LC	1 (20)
Exotic position in LS	4
From the base of LS in the superior mediastinum	1 (25)
Common trunk of left VA & left inferior thyroid artery	1 (25)
Distal to thyrocervical trunk	1 (25)
High at LS	1 (25)
Unknown	16 (1.6)
Right VA	145 (12.5)
Arch	94 (64.8)
RS root	72 (76.6)
Distal to LS	19 (20.2)
Between RC & RS (right aortic arch)	1 (1.1)
Proximal to LS	1 (1.1)
Proximal to LS (right aortic arch)	1 (1.1)
Extra-arch	47 (32.4)
RC	40 (85.1)
Brachiocephalic trunk	3 (6.4)
Descending aorta (distal to the aberrant RS)	1 (2.1)
Ascending aorta	1 (2.1)
Right external carotid artery	1 (2.1)
Thyrocervical trunk	1 (2.1)
Exotic position in RS	3 (2.1)
RS (distal to the right thyrocervical trunk)	2 (66.7)
Common trunk of right VA & right inferior thyroid artery	1 (33.3)
Unknown	1 (0.7)
Bilateral (left VA/right VA)	32 (2.8)
Arch (between LC & LS)/RC	15 (46.9)
Arch (between LC & LS)/arch (distal to LS)	7(21.9)
Arch (between LC & LS)/brachiocephalic trunk	2 (6.3)
Arch (between LC & LS)/arch (between LC & LS)	1 (3.1)
Arch (between LC & LS)/descending aorta	1 (3.1)
Arch (?)/arch (?)	1 (3.1)
Arch (between LC & LS)/iunction of RB & RS	1 (3.1)
Arch (LS root)/brachiocephalic trunk	1 (3.1)
Left VA + LS/RC	2 (6.3)
Left internal carotid artery/right internal carotid artery	1 (3.1)

LC=left common carotid artery; LS=left subclavian artery; RC=right common carotid artery; RS=right subclavian artery; VA=vertebral artery

**Table 4:** Location of dual origins of vertebral artery.

Location of dual origins	n (%)
Left VA	30 (56.6)
Arch (between LC & LS) + LS	25 (83.3)
LS + LS	2 (6.7)
Arch + arch (both between LC & LS)	1 (3.3)
LC + LS (aberrant)	1 (3.3)
Unknown	1 (3.3)
Right VA	15 (28.3)
RS + RS	15 (100)
Dual aberrant origins of right VA + single aberrant origin of left VA	3 (5.7)
Arch (between LC & LS); RS + RS	2 (66.7)
Unknown	1 (33.3)
Bilateral dual origins	5 (9.4)
RS + RS; arch + LS	2 (40)
Arch (between LC & LS); RS + brachiocephalic trunk	1 (20)
RS + RS; LS + LS	1 (20)
RS + RS; arch + arch	1 (20)

LC=left common carotid artery; LS=left subclavian artery; RS=right subclavian artery; VA=vertebral artery

**Fig. 1:**
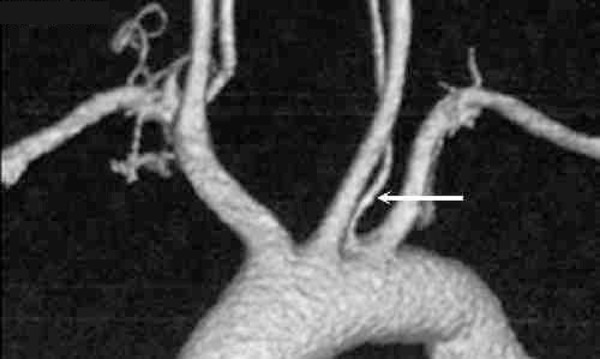
Aortogram shows a 4-vessel aortic branch pattern with a sequence of brachiocephalic trunk, left common carotid artery, left vertebral artery (arrow) and left subclavian artery from left to right.

Aortic arch branching was stated in 1270 (98.7%) cases, including 1 (0.1%) 2-vessel, 236 (18.6%) 3-vessel, 1011 (79.7%) 4-vessel and 21 (1.7%) 5-vessel aortic arch branching patterns. The brachiocephalic trunk-left common carotid artery-left VA-left subclavian artery pattern was the most common 4-vessel arch branching pattern accounting for 84.8% (Figure 1). Two hundred and eleven common trunks of the arteries were found in 207 (16.1%) cases with one common trunk in 203 (98.1%) patients and two common trunks in 4 (1.9%). The bovine trunk (*i.e.*, the common trunk of the brachiocephalic trunk and left common carotid artery) was the most frequent arterial trunk seen in this cohort.

A total of 115 (12.0%) patients had one or more congenital cardiovascular anomalies, where aberrant right subclavian artery was the most common (55.7%). In the patient group, 89 (9.3%) patients had one or more acquired vascular associations, including aneurysms, obstructions, stenosis, thrombus formation and dissection, *etc.*, 18 (20.2%) of which were VA-associated lesions and 8 (0.8%) having a VA dissection.

There were 43 (4.5%) patients with cerebrovascular lesions, including cerebral infarcts^[9-24]^ in 17 (39.5%), cerebral vascular aneurysms without cerebral hemorrhage^[14,23,25,26]^ in 4 (9.3%) and cerebral hemorrhage in 18 (41.9%), and ischemic cerebrovascular disease^[25]^, transient ischemic attack^[14]^, partial thrombus detected in the left internal jugular vein and sigmoid sinus^[27]^, and vertebrobasilar insufficiency^[28]^ in 1 (2.3%) patient each. The 18 cerebral hemorrhages were subarachnoid hemorrhage in 14 (77.8%)^[29-39]^ (8 were associated with an aneurysm of the cerebral artery^[29-31,33,35-37,39]^), intraventricular hemorrhage in 2 (11.1%)^[40,41]^ and intracerebral hemorrhage^[42]^ and subarachnoid and intraventricular hemorrhages^[43]^ in 1 (5.6%) patient each.

The cardiovascular, cerebrovascular, or orthopedic complications, accounting for 84.8% (78/92), 13.7% (13/95) and 4.2% (4/95), respectively, were described along with their management in 95 (9.9%) patients. There were no significant differences in the curative ratio and mortality between the cardiovascular and cerebrovascular groups (curative ratio: 84% *vs.* 63.6%, *χ*^2^=1.8, *P*=0.176; mortality: 4% *vs.* 0%, *χ*^2^=0.5, *P*=0.501). The curative ratios did not show any significant differences between the surgically, conservatively and interventionally treated patients of the cardiovascular group (80% *vs.* 80% *vs.* 100%, *χ*^2^ = 1.2, *P*=0.551) (Table 5).

**Table 5:** Clinical outcomes of cardiovascular and cerebrovascular complications subjected to various management strategies, n (%).

Management	Cardiovascular					Cerebrovascular				
	Cured	Improved	No change	Died	NG	Cured	Improved	No change	Died	NG
Surgical	12 (21.4)	1 (1.8)	1 (1.8)	1 (1.8)	41 (73.2)	1 (33.3)	1 (33.3)			1 (33.3)
Conservative	4 (28.6)		1 (7.1)		9 (64.3)	2 (33.3)	2 (33.3)	1 (16.7)		1 (16.7)
Interventional	5 (83.3)				1 (16.7)	2 (66.7)				1 (33.3)
Hybrid					1 (100)					
Y-knife						1 (100)				
Exercise program					1 (100)					
Follow-up						1 (100)				

NG=not given

Logistic regression analysis showed significant regressions between bovine trunk and left VA (*P*=0.000) of the whole setting, and between the dual origins of VAs and cerebral infarct/thrombus (*P*=0.041), between associated alternative congenital vascular variants and cervical/aortic dissection/atherosclerosis (*P*=0.008) of the patient cohort. No correlation was found between associated alternative congenital vascular variants and VA dissection/stenosis (*P*=0.792) or between dual origins of VA and female gender (*P*=0.788).

Multiple logistic regression demonstrated that side of the aberrant origin of VA (left VA) (*P*=0.014), arch branch pattern (direct arch origin) (*P*=0.019), presence of the common trunk (*P*=0.019), associated acquired vascular disorder (*P*=0.034) and the patients who warranted management (*P*=0.000) were significant risk predictors for neurological sequelea; while gender (*P*=0.512), patient's age (*P*=0.069), aberrant origin of VA (*P*=0.075), hypoplasia of VA (*P*=0.669) and abnormality of VA (*P*=0.944) were not.

## DISCUSSION

### Incidence

Direct aortic origin of left VA is the most frequent anatomic variant of VA with a prevalence of 2.4-5.8% in several large autopsy series^[44]^, 2.4-2.5% in patients for cerebral angiography for different reasons^[4,45]^ and 5.25% in patients with suspected extracranial cerebrovascular disease by selective 4-vessel angiography^[46]^. Aberrant right VA is an extremely rare anomaly^[47]^. Ding et al.^[48]^ reported a cadaver series of 12 VAs with an aberrant origin, which were all presented on the left, arising from the aortic arch (83.3%) (75% of which originated from the arch between the left common carotid and left subclavian arteries), left common carotid (8.3%) and left external carotid arteries (8.3%). Tsai et al.^[49]^ described, in patients with an aberrant right subclavian artery, the overall prevalence of left VA anomaly was 5.9% (6/102) and that of right VA anomaly was 13.7% (14/102). However, the true incidence of an anomalous origin of right VA from the right common carotid artery remains unknown^[50]^. With reference to the circumference of the subclavian artery, the location of the origin was cranial in 47%, dorsal in 44%, caudal in 6% and ventral in 3% with an even distribution between the cranial and dorsal quadrants^[51]^.

### Embryology

The VAs develop between 33 and 55 days during intrauterine life. The VA is normally formed by the longitudinal anastomoses linking the 7 cervical intersegmental arteries. The intersegmental arteries obliterate soon except for the 7^th^ intersegmental artery, which develops into the subclavian artery involving the origin of VA. In a few cases, the anastomosis between the 6^th^ and 7^th^ intersegmental arteries does not develop on the left side and the 6^th^ intersegmental artery remains, and then the left VA is arising from the aortic arch between the left common carotid and subclavian arteries. Cranial migration of the right VA can result in its branching directly of the aortic arch, and migration relative to the right thyrocervical trunk can lead to some of the other variants^[52]^. Origin of VAs from the aorta suggests that part of the aortic arch arises from the left 7^th^ intersegmental arteries or there was increased absorption of embryonic tissue of the left subclavian artery between origin of aortic arch and the VA^[53]^. A faulty degeneration of the primitive dorsal aorta and two intersegmental arteries is considered to be responsible for the development of duplicate VAs^[54]^.

### Anatomy

Normally, the VA starts above the first rib plane, accounting for 97.1% (99/102) while in a few cases, its origin was below the first rib plane but in the thorax instead accounting for 2.94% (3/102) ^[48]^. Meila et al.^[25]^ reported that 94.2% of left VA originated from left subclavian artery and entered the *foramen transversarium* at C6 in nearly all cases; and 6.3% of left VA originated from the aortic arch and entered the *foramen transversarium* either at C4, C5 or C7 but never at C6. Uchino et al.^[23]^ noted that most left VAs with direct aortic origin proximal to the left subclavian artery entered C4 or C5, and all left VAs with direct aortic origin distal to the left subclavian artery entered C7. All right VAs with proximal right subclavian origin entered C5, C4, or C3; whereas the aberrant right VA entered C7. Moreover, the duplicated segments of left VA fuse at the C5-6 level into a single VA, which then enters the *foramen transversarium* of C5^[3,55,56]^.

Dodevski et al.^[57]^ reported that the VAs on both sides were equal in diameter in 23.3% patients. The right VA was larger in 30% patients, and the left VA was larger than the right in 46.6% patients. Hypoplasia of VA was found in 6.67% patients. In two patients hypoplasia was on the right side, in one patient on the left side, and in one patient bilateral hypoplasia of the vessel on both sides. Matula et al.^[58]^ stated a hypoplastic artery diameter <3.5 mm was found in 16 (6.96%) cases. Trattnig et al.^[45]^ reported 4.78% of hypoplasia was found on the right side and 2.17% on the left side. Patients with hypoplastic VA may have a high probability of posterior circulation stroke, with atherosclerotic susceptibility and ipsilateral lesions in the VA territory^[59]^.

The significance of the common trunk of the arteries has not been properly indicated in the literature. In 1967, Mueller & Hinck^[60]^ described one patient with bilateral subclavian artery obstruction occurring distal to the origin of VAs. Both VAs supplied to the thyrocervical trunks via extensive collateral vessels, which was termed as "thyrocervical steal". The incidence of a common trunk of the VA and thyrocervical trunk originating from the subclavian artery was 0.58%, and the incidence of a common trunk of the right and left VAs and inferior thyroid artery were 0.64% and 0.13%, respectively^[37]^. Ding et al.^[48]^ presented a case of cadaver whose left VA originated from the left external carotid artery forming a common trunk with the occipital and posterior auricular arteries.

The frequency of duplication of the VA has been identified in 0.72% of cadavers^[19]^. Prevertebral duplication may occur when a portion of the primitive dorsal aorta persists along with two intersegmental vessels connected to the true VA^[61]^. Another explanation is a failure of the fifth or sixth intersegmental artery to regress, which adds a further origin to the VA along with the normal 7^th^ segment^[55]^. On the right side, both segments usually derive from the right subclavian artery. On the left side, the lateral crus of the duplicated artery commonly starts from the left subclavian artery and the medial one from the aortic arch, between the left common carotid artery and the left subclavian artery. There were also reports of duplicated vessels derived from the thyrocervical trunk^[62]^. When one of the duplicated origins connects with the contralateral VA and the other ends more distally, opens into the basilar artery. The duplication of the VA might be connected with morphological changes of the VA wall^[63]^. A duplicated VA is a significant predisposing factor of vertebrobasilar cervical artery dissection due to local histological defects or significant hemodynamics alterations^[10,64]^. Clinically, patients with diagnosed VA duplication can present a variety of symptoms such as vertigo, dizziness or occipital heaviness. A probable explanation for this is that the lumen of the duplicated vessels can be decreased, predisposing it to easier kinking, resulting in posterior circulation insufficiency^[65]^. The VA can be easily damaged during severe cervical spine injuries with rapid subluxation, deceleration, fracture through the *foramen transversarium*, or flexion of the cervical spine, *i.e.*, the VA is easy to suffer from trauma, contusion and crushes as a result of cervical spine injuries^[66]^.

Metrology of the vessels demonstrated that the length of the prevertebral segment of the aberrant left VA was 88.5 mm, and the length of the prevertebral segment of the right VA was 44.3 mm^[67]^. Aortic arch branch measurements showed that left VA had a smaller diameter but longer distance from the mid-vertebrae line (Table 6).

**Table 6:** Aortic arch branch measurements^68^

Vessel	Diameter, mean (range) (mm)	Distance from mid-vertebrae line, mean (range) (mm)
BT	18 ± 3.9	9.3 ± 4.7 (0-20)
LC	9.8 ± 1.9 (6-15)	9.9 ± 5.3 (1-20)
Left VA	5.5 (5-6)	26.5 (22-31)

BT=brachiocephalic trunk; LC=left common carotid artery; VA=vertebral artery.

In only one (11%) cadaver the left VA arose with the left subclavian artery from a common trunk. The trunk originated from the arch behind the left common carotid artery. The diameter of the trunk was 20.0 mm. The distance from its origin to the mid-vertebrae line was 31 mm^[68]^. The mean distance between the brachiocephalic trunk and left common carotid artery was 0.1-0.5 cm and between the left common carotid artery and left subclavian artery was 0.3-2.0 cm^[69]^. Some authors tried to depict the anatomy of the dual originated VAs, where the medial limb (3.9 mm in diameter at its origin) originated from its orthodox position, whereas the lateral limb (2.4 mm in diameter at its origin) arose more posteriorly close to the origin of thyrocervical trunk^[56]^.

### Diagnosis

Color Doppler sonography is the firstline imaging modality for evaluation of the VAs, although the origin cannot be visualized with this modality in a significant number of patients. Non-visualization of color signals and the absence of spectral tracings will easily establish a diagnosis of occlusion in the extracranial VA segment. In previously reported cases, the diagnosis of a duplicate origin was proven by angiography or, in 1 case, by angiography and magnetic resonance imaging^[70]^. An abrupt change in the diameter of the VA raised the possibility of a dissection^[71]^. Diagnostic problems may ensue when a filling defect is created at the junction between the anomalous origin and the normal origin of the VAs from non-opacified blood. An abrupt change in the size of VA at its juncture with a dual origin would suggest hypoplasia or even pathological narrowing of the vessel. With the increasing utilization of digital vascular imaging by way of the venous route, and with a smaller field of view, one may consider the VA to be hypoplastic or even occluded unless the possibility of a bifid origin is considered^[31]^.

### Clinical Significance

The patients with left VA variants are usually asymptomatic, or with symptoms resulting from other than the aberrant VA. Rare cases have presented with dizziness, but this does not seem to be associated with the anomalous origin^[5]^. The patients may be asymptomatic unless the VA is involved by atherosclerotic lesions^[72]^. The left VA origin anomaly with its C4 entrance and contralateral hypoplasia could cause ataxia during head rotation^[5]^. Symptoms of patients with duplicated VA are probably not related to these anomalies of VAs^[62]^. The significance of recognizing left lateral medullary infarction associated with mild intracranial VA stenosis^[22]^. The anomalous VA origin may be an independent risk factor for arterial dissection; the longer extra-cranial course may lead to increased vulnerability of the vessel wall to shear stress resulting in intimal tear and dissection^[11]^. Headaches and neurological symptoms in a patient with an anomalous VA origin should initiate a thorough investigation for arterial dissection^[11]^.

Komiyama et al.^[4]^ in their study detected arterial dissection in 17 patients with an incidence of 1.9%. They analyzed that the left VA of aortic origin showed a remarkably higher incidence of arterial dissection than left VA of a left subclavian and right VA of a right subclavian artery origin. The reasons for the high incidence of arterial dissection associated with VA of aortic origin remain to be elucidated. An anomalous left VA arising directly from the aortic arch typically enters the C4 or C5 *foramen transversarium*, resulting in a longer course of VA in the neck, thus predisposing to VA dissection. The present study revealed an incidence of VA dissection of 0.9% (9/955).

VA morphological variants determine regional hemodynamic solution^[66]^. The VA blood flow volume accounts for 31% of the total brain flow volume at the age of 4 years, and declines significantly thereafter until the age of 18 years. After this age the blood flow volume percentage of VAs stabilizes at 24%^[66]^.

There was no association between a bovine trunk and direct origin of left VA^[73]^. Also, there was no evidence of association between a dual left VA origin and distal vertebral thromboembolism, or between other vascular anomaly and premature atherosclerosis or intracranial dissection^[22]^. The incidence of duplicated VA among the patients for computed tomography angiography was 0.74%, all occurring in female patients^[25]^. Contrary to the above-mentioned, the present study revealed close relations between bovine trunk and left VA, dual origins of VAs and cerebral infarct/thrombus formation, and between associated alternative congenital vascular variants and cervical/aortic dissection/atherosclerosis, but no female gender predilection for dual origins of VA. Moreover, aberrant origin of left VA and aberrant origin of right subclavian artery were frequent in Down syndrome patients. In return, aberrant origin of left VA and right subclavian artery might be helpful in the diagnosis of Down syndrome^[74]^.

The usual procedures of endarterectomy of the proximal left VA or even transplantation to the left common carotid artery may not be necessary if the true anatomic configuration is identified^[31]^. The isolated left VA was reconstructed with a saphenous vein graft interposed between the native isolated left VA and the side of the graft branch anastomosed to the left subclavian artery^[6]^.

The patient management using an antiplatelet regimen is an alternative for these patients^[75]^. As an aortic arch surgery might be complicated by ischemic issues, which can be caused by unrecognized variation of its vascular anatomy^[68]^, accurate assessment of anatomic variations of VAs is recommended before aortic arch surgery or endovascular interventions^[5]^. Care has to be taken when planning to cover the origin of left subclavian artery^[76]^. Detailed knowledge of an anomalous origin of supraaortic arteries is also of importance for patients who have to undergo 4-vessel angiography^[77]^. A duplicated VA influences surgical procedures performed on the head and neck region. Visualization of only one trunk of a double VA during catheterization can lead to misdiagnosing the VA as hypoplastic^[78]^. During surgical incision of the muscles of the transverse spinal processes (deep cervical region) one may damage an abnormally long prevertebral VA segment (V1)^[66]^.

## CONCLUSION

The patients with neurological symptoms and those for neck and chest operations/interventions should be carefully screened for the possibility of an aberrant origin of VA. The results from the cadaver metrology study are very helpful in the design of the aortic stent. The arch branch pattern has to be taken into consideration before any maneuver in the local region so as to avoid unexpected events in relation to aberrant VAs.

**Table:** 

**Author's roles & responsibilities**	
SMY	Study conception and design; analysis and/or interpretation of data; manuscript writing, final approval of the manuscript
